# Comut-viz: efficiently creating and browsing comutation plots online

**DOI:** 10.1186/s12859-023-05351-8

**Published:** 2023-06-01

**Authors:** Qiaonan Duan, Weiyi Wang, Feiling Feng, Xiaoqing Jiang, Hao Chen, Dadong Zhang, Tongyi Zhang

**Affiliations:** 1 Department of Translational Medicine, 3D Medicines Inc., Shanghai, China; 2 General Surgery Department, Huadong Hospital Affiliated to Fudan University, Shanghai, China; 3 Department of Biliary Tract Surgery I, Third Affiliated Hospital of Naval Medical University (Eastern Hepatobiliary Surgery Hospital), Shanghai, China

**Keywords:** Visualization, Comutation plot, Genomic analysis, Mutation, Web server

## Abstract

**Background:**

Comutation plot is a widely used visualization method to deliver a global view of the mutation landscape of large-scale genomic studies. Current tools for creating comutation plot are either offline packages that require coding or online web servers with varied features. When a package is used, it often requires repetitive runs of code to adjust a single feature that might only be a few clicks in a web app. But web apps mostly have limited capacity for customization and cannot handle very large genomic files.

**Results:**

To improve on existing tools, we identified features that are most frequently adjusted in creating a plot and incorporate them in Comut-viz that interactively filters and visualizes mutation data as downloadable plots. It includes colored labels for numeric metadata, a preloaded palette for changing colors and two input boxes for adjusting width and height. It accepts standard mutation annotation format (MAF) files as input and can handle large MAF files with more than 200 k rows. As a front-end only app, Comut-viz guarantees privacy of user data and no latency in the analysis.

**Conclusions:**

Comut-viz is a highly responsive and extensible web app to make comutation plots. It provides customization for frequently adjusted features and accepts large genomic files as input. It is suitable for genomic studies with more than a thousand samples.

**Supplementary Information:**

The online version contains supplementary material available at 10.1186/s12859-023-05351-8.

## Background

Comutation plot [1] represents mutation data as a matrix of tiles with the rows being mutated genes and the columns being sequenced samples. It is widely used in genomic studies to visualize the mutation landscape. Several bioinformatics packages [2–4] have been developed to create the plots. These packages are largely language specific and not interactive, requiring repetitive runs to adjust a single feature. Interactive web tools have been developed to make the process more efficient. They include standalone web apps like CoMutPlotter [5] and jsComut [6], and more comprehensive platforms like cBioPortal [7] and Oviz-Bio [8]. CoMutPlotter generates static images with no customization. JsComut require users to manually click an update button when changing the appearance of an existing plot. It lacks colored labels for continuous data and the ability to modify width and height. The cBioPortal platform has the OncoPrinter module to generate comutaton plots but allows no color customization and height adjustment. The Oviz-Bio platform is a newly developed platform with more interactive features. But it is not open-sourced and accepts only a non-standard file format as input. This limits its use and adaption by the wider community of bioinformatics developers. Unsatisfied with current solutions, we developed Comut-viz, a highly responsive open-sourced web app that simplifies the steps in making a comutation plot.

## Implementation

Considering the privacy and the security of mutation data, we designed Comut-viz as a front-end only app: a user’s input data is processed locally within a user’s browser and never sent to a back-end server. A schematic flow chart illustrating the implementation was provided in the supplementary (Additional file 1: Fig. S5). React 18 was used as the front-end framework to orchestra the app. It implements three views: an input view to submit data, a filter view to the customize data and a visualization view to draw on the data.

A JavaScript (JS) ECMAScript6 (ES6) class named ComutData was developed to transform input tables into JS objects rendered in the visualization view. It implements waterfall sorting commonly used in organizing samples. In the visualization view, ES6 classes were built for each component of the plot including the grid, the colored labels, the bar plots and the legends. They share the same interface and can be easily arranged in a scalable vector graphics (SVG) container. A color picker was developed to customize colors on the plot including the base color of a gradient legend. The base color of a gradient legend was mapped into HSL values with the hue (H) and saturation (S) fixed and the lightness (L) mapped to the input continuous values.

## Results

### The input view

**Fig. 1:**
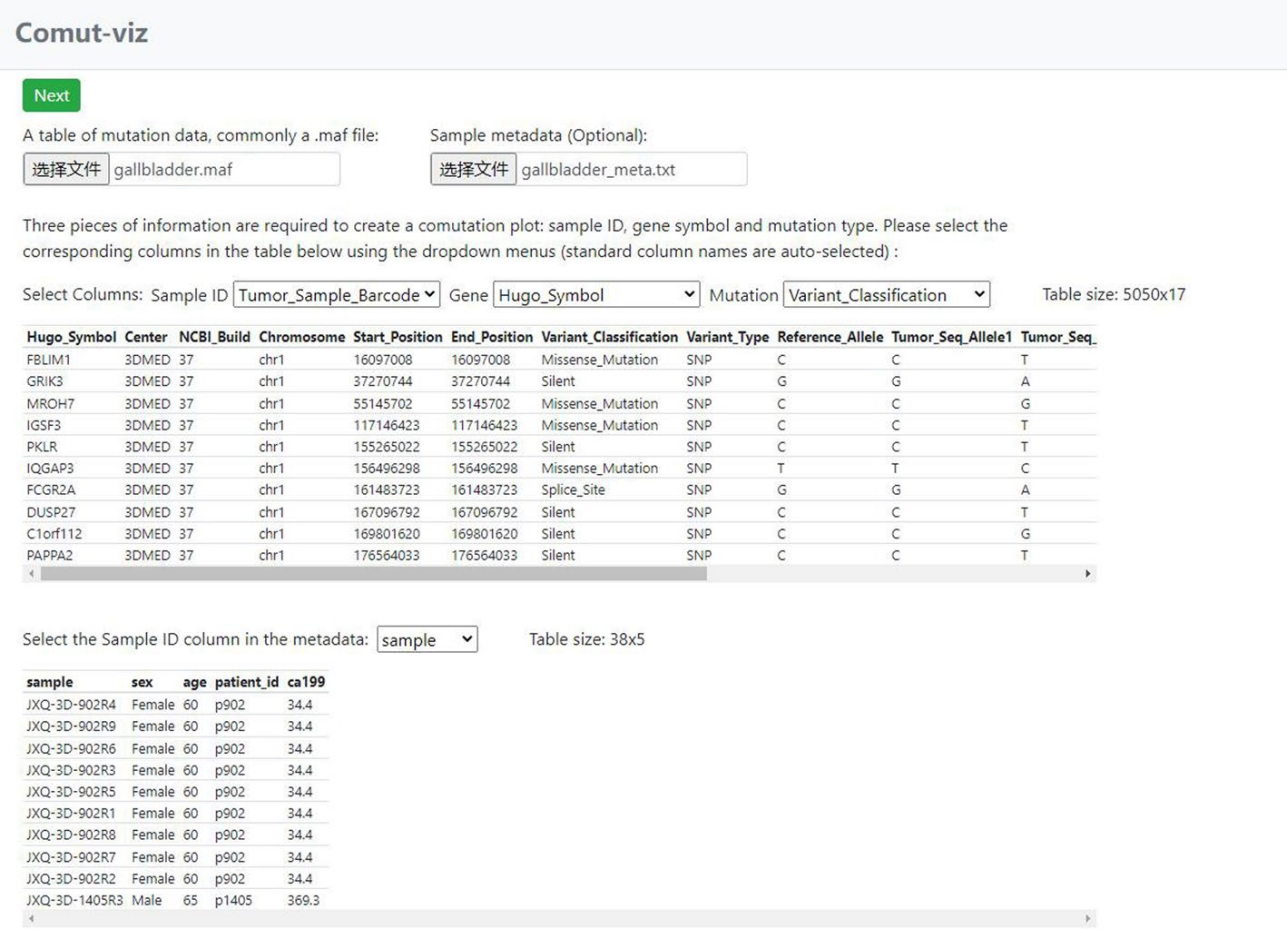
A screen shot of the input view. It shows the input view with the “example 5 k” data and metadata loaded. The sample, gene and mutation type columns are automatically selected

In the input view (Fig. 1), users upload a table of mutation data in a standard MAF file or a tab/comma delimited text file. The app will display a preview of the table with the first 10 rows. Three select elements will appear above the preview to let users choose the sample IDs, gene and mutation type columns in the table. They will be automatically selected if the input file is a standard MAF file. After selection, click on the Next button to navigate to the filter view. Users can optionally upload a table of sample metadata in a tab/comma delimited text file as the second input. The metadata must include an ID column containing the same sample IDs as in the mutation data. A similar preview will be displayed for the metadata. A select element will appear above the metadata preview to let users choose the sample column. By default, the first column is selected as the sample column.

Users can test the app by clicking on the example buttons. The “example 5 k” button loads a tab delimited text file that contains mutation profiles of patient-derived primary cancer cell lines collected from different regions of gallbladder carcinoma tumors from seven patients [9]. Its associated metadata includes patient ID, age, gender and serum Carbohydrate antigen 19-9 (CA19-9) levels. The “example 141 k” button loads a MAF file with 141 k rows that are mutation profiles of the TCGA-LUSC cohort [10].

### The filter view

**Fig. 2:**
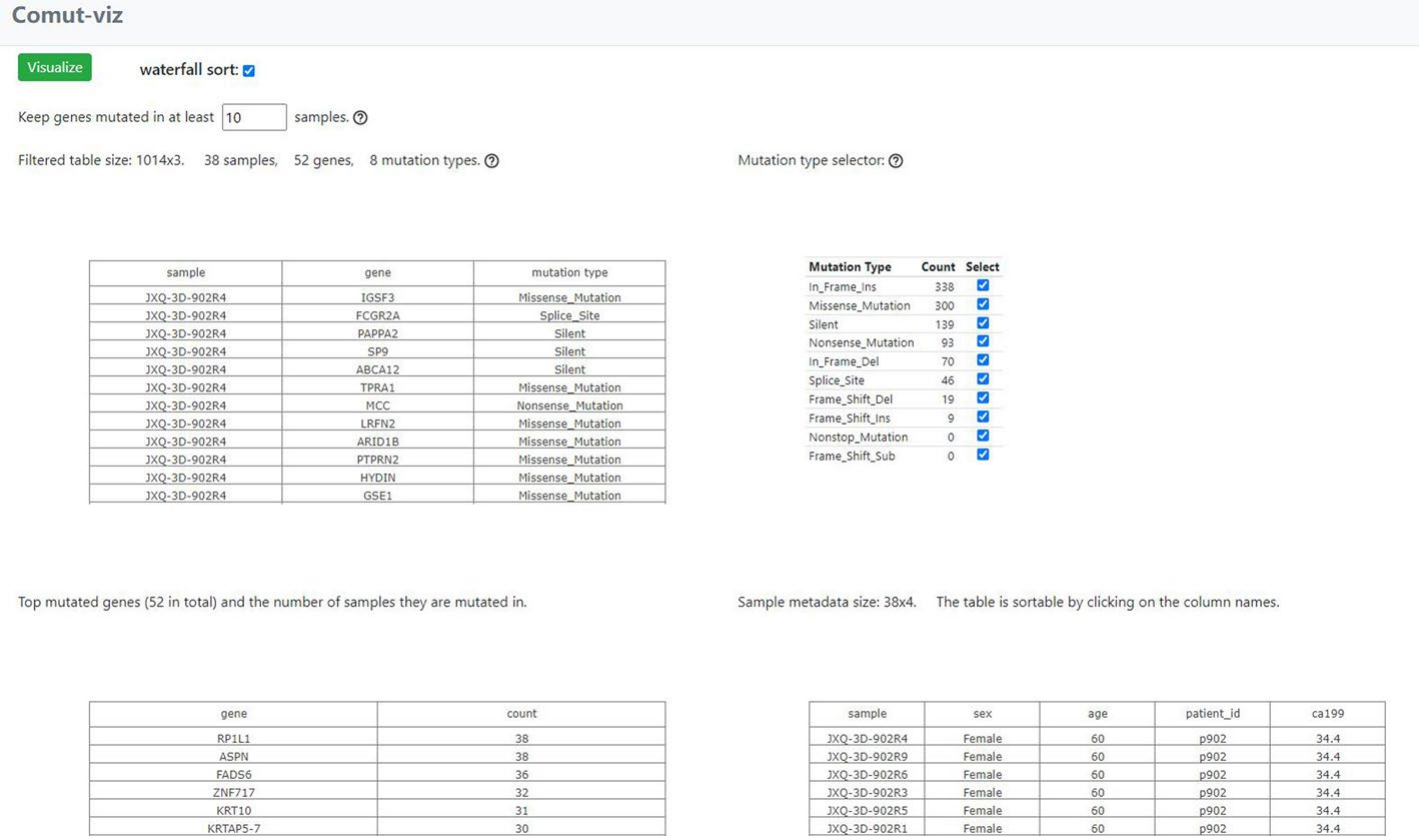
A screen shot of the filter view. It shows the filter view of “example 5 k”

In the filter view (Fig. 2), the app display three tables: the filtered mutation table, a table of mutation types and a table of top mutated genes. In case the mutation table contains too many genes to visualize, the app provides an option to filter them by sample count. Summary statistics on top of each table are updated with each filtering. If a metadata table is provided, it will be also displayed in this view. The columns of the metadata table will be parsed at this stage according to the inferred data types. Columns of string type will be treated as categorical labels and those of numeric type as gradient labels.

By default, the filter view keeps at most 60 top mutated genes. Users can change the number of top mutated genes by adjusting the sample count filter. When the top mutated genes are less than or equal to 60, the waterfall checkbox is automatically checked. The app will sort the samples by waterfall sorting in the visualization view. When waterfall checkbox is unchecked and the metadata table provided, the app will sort the samples by the order in the metadata. This mechanism allows users to use the metadata to specify the sample order in the final visualization.

Users can use the checkboxes in the mutation type table to remove mutation types they do not want to show in the comutation plot. Uncheck a mutation type may cause the counts of other mutation types to change. The number of genes passing the current threshold may also change. This is because the reduced number of mutation types decreases the qualified mutations counted for each gene. Click on the Visualize button to navigate to the visualization view.

### The visualization view

**Fig. 3:**
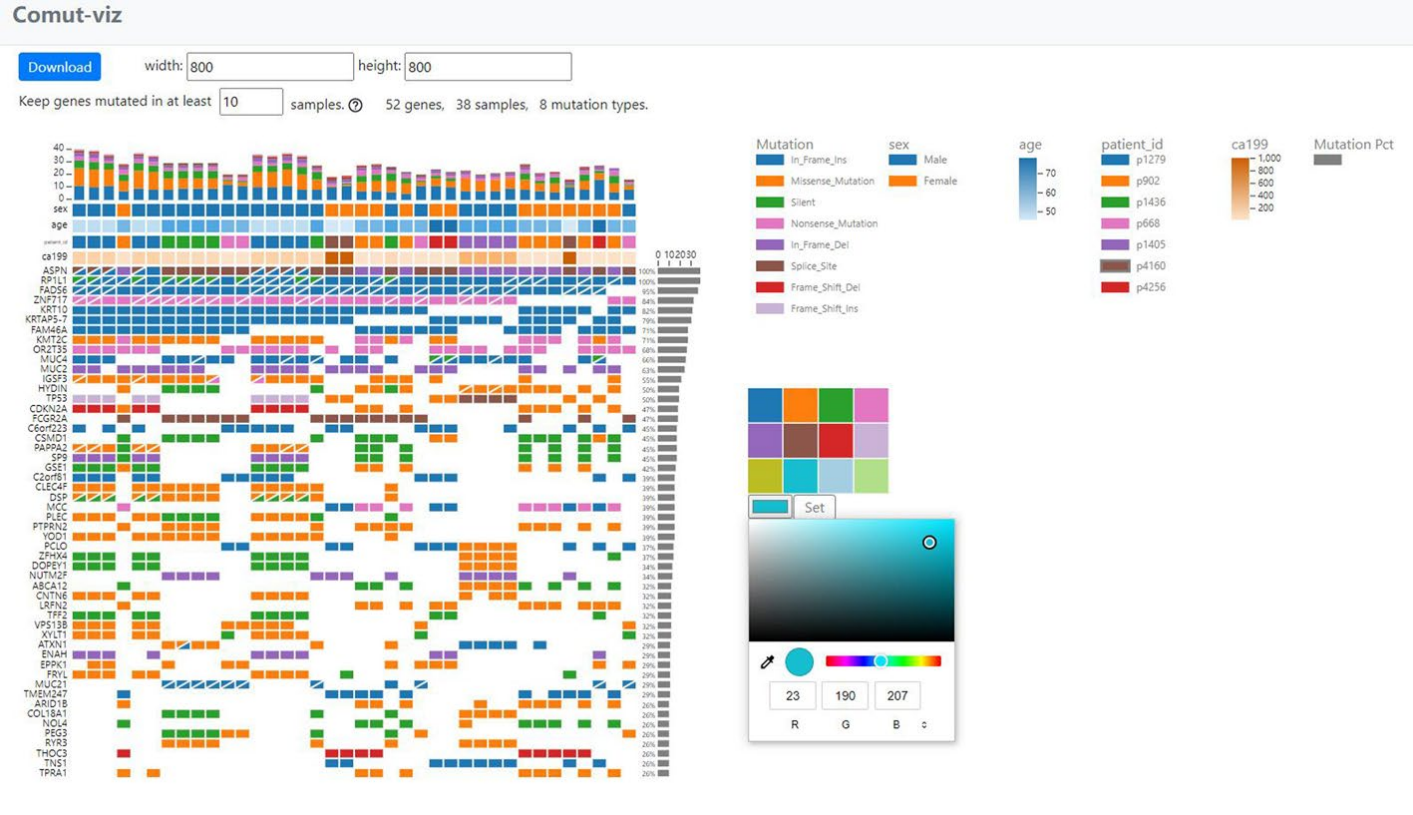
A screen shot of the visualization view. It shows the visualization view of “example 5 k”. The color and height of the plot have been adjusted

The visualization view (Fig. 3) consists of three regions: the control and plotting regions on the left and the legend region on the right. In the control region, there are two input boxes to control width and height of the figure and a filter to fine-tune the number of genes displayed in the figure. In the plotting region, a bar plot is placed on top to show the number and types of mutations in each sample. A side bar chart on the right reveals the percentage of samples a gene is mutated in. If metadata is provided, colored labels will be displayed between the bar plot and the grid. They will be categorical if the data is of string type and gradient if of numeric type. Mouse-over on any shape will pop up detailed information. Mouse-over on a tile will also invoke black boxes highlighting its metadata on the X and Y axes.

Legends are created for each colored component. Clicking on a legend opens up a color picker that is preloaded with 12 distinct colors for quick selection. Users can create a customized color by clicking on the color box in the picker. All text labels are auto-scaled when the size of the figure is changed. Clicking on the “Download” button downloads the plot and the legend as two separate SVG figures that can be edited in vector graphics editors such as Adobe Illustrator or Inkscape.

Besides the two examples provided in the app, we also tested Comut-viz on a large MAF file with 229 k rows (~ 33 MB). We tested it on a Windows desktop with an I5-10,400 CPU and 16 GB memory. The MAF file can be found in the Github repository and is a concatenation of the mutation data from the TCGA-LUSC and TCGA-BRCA studies.

## Conclusions

We developed Comut-viz to make creation of comutation plots more efficient. We found filtering genes and changing colors the two most repetitive tasks in fine-tuning a final figure. Comut-viz implements two filters and a preloaded color palette to address that. Built with the React framework, it is instantly responsive to user manipulations. Developed using the object-oriented ES6 syntax, Comut-viz is a collection of reusable components and classes that are readily exported to other projects. Users can easily integrate Comut-viz into their existing data analysis pipeline and make the visualization process more efficient.

## Availability and requirements

Project name: Comut-viz. Project home page: https://frlender.github.io/comut-viz-app/ (running instance). https://github.com/frlender/comut-viz (development repository). Operating system(s): Platform independent. Programming language: JavaScript. License: MIT license. Any restrictions to use by non-academics: None.

## Supplementary Information

**Additional file 1**. Detailed illustration of the many components of Comut-viz. The file provides detail illustration of the many components of Comut-viz in three views. It also provides a flow chart to illustrate how Comut-viz works on a high level.

## Data Availability

The “example 5 k” data and metadata can be found at (https://github.com/frlender/comut-viz/blob/main/public/gallbladder.maf) and at (https://github.com/frlender/comut-viz/blob/main/public/gallbladder_meta.txt). The “example 141 k” data can be found at (https://github.com/frlender/comut-viz/blob/main/public/TCGA-LUSC.maf). The large MAF file with 229 k rows can be found at (https://github.com/frlender/comut-viz/blob/‌main/public/TCGA-LUSC-BRCA.maf).
