# Supplementary material for: Comut-viz: efficiently creating and browsing comutation plots online
Source: BMC Bioinformatics. 2023 Jun 1;24:226. doi: 10.1186/s12859-023-05351-8 (PMC10236844; doi:10.1186/s12859-023-05351-8)
Supplement: Supplementary file 1 — Additional file 1. Detailed illustration of the many components of Comut-viz. The file provides detail illustration of the many components of Comut-viz in three views. It also provides a flow chart to illustrate how Comut-viz works on a high level. [file 12859_2023_5351_MOESM1_ESM.pdf]

The Input View

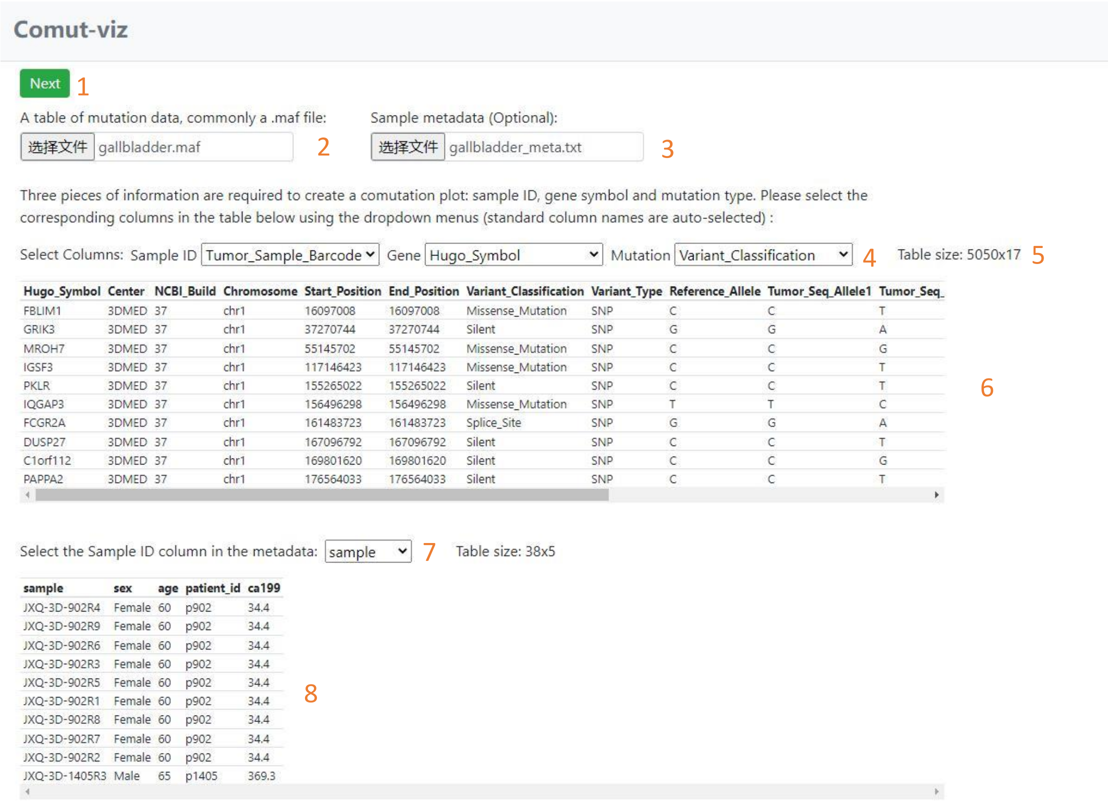

Figure S1. The input view with the “example 5k” files manually uploaded.

The entry point (Figure 1) to the app is to upload a text file of mutation data (2). Normally, it should be a MAF file containing mutation data of multiple samples. A tab/comma delimited text file containing the same information is also supported. Example files can be loaded by clicking on the example buttons. After the mutation data is loaded as a table, a preview of it will be shown with the first 10 rows (6). The size of the table is displayed on top right (5). To draw a comutation plot, we only need three columns: sample, gene and mutation type. But usually the input file has many more columns. So we provide users with three select elements to choose the columns (4). The options in the three select elements are mutually exclusive, meaning that if one column is selected in an element, it could not be selected in another. It helps to prevent users from accidentally select one column twice.

Only after three columns are selected will the next button (1) be enabled. Clicking on it navigates to the filter view. The next button will only appear after the mutation file is loaded or the example button is clicked.

Optionally, users can upload a tab/comma delimited table of sample metadata (3). The metadata must have one column containing the same sample IDs as in the mutation data. A similar preview of the table with the first 10 rows will be shown (8). A select element will appear above the preview to let users choose the sample ID column (7). By default, the first column is selected as the sample ID column. Click on the “example 5k” button (4) will load the example metadata at the same time. The example buttons will disappear if users have uploaded either a mutation data file or a metadata file.

The Filter View

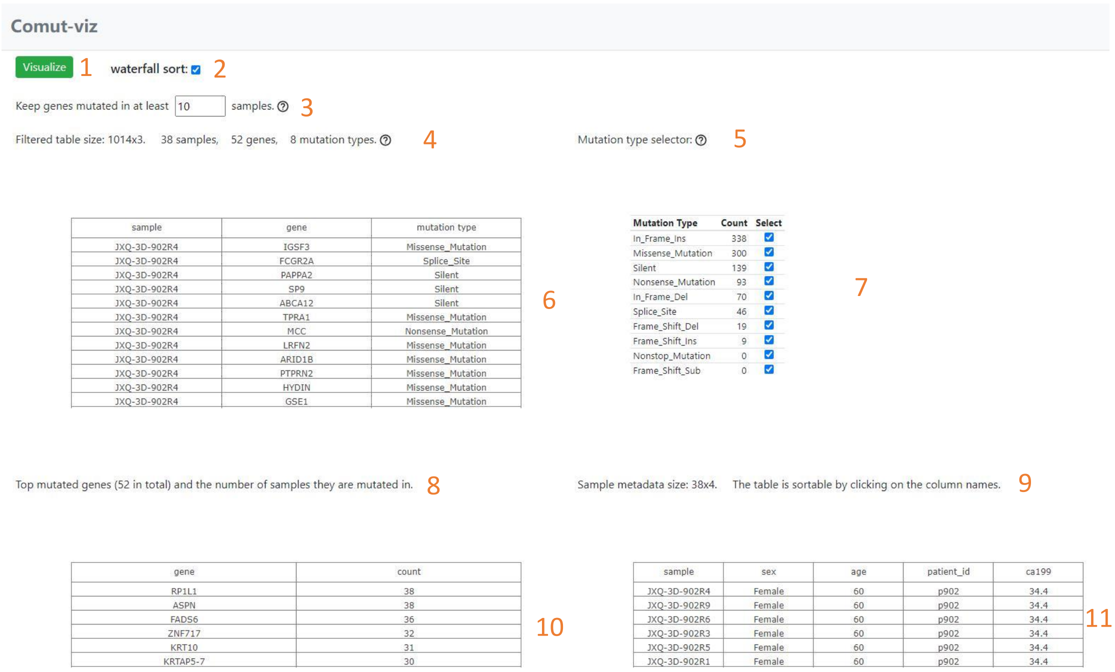

Figure S2. The filter view with the “example 5k” data loaded.

In the filter view (Figure 2), the app display three tables: the filtered mutation table (6), a table of mutation types (7) and a table of top mutated genes (10). In case the mutation table contains too many genes to visualize, the app provides an option to filter them by sample count (3). Summary statistics on top of each table is updated with each filtering (4, 8). If the metadata table is provided, it will be also displayed in this view (11) along with its summary statistics (9).

By default, the filter view selects a sample count threshold (3) that keeps at most 60 top mutated genes. When the top mutated genes are less than or equal to 60, the waterfall checkbox (2) is automatically checked. The app will sort the samples using waterfall sorting in the visualization view. When waterfall checkbox is unchecked and the metadata table is provided, the samples will be sorted in the order as in the metadata. This mechanism allows users to use metadata to specify the order of samples in the final plot. Clicking on the Visualize button (1) navigates to the filter view.

Users could use the checkboxes in the mutation type table (7) to remove mutation types they do not want to visualize in the comutation plot. Uncheck a mutation type may cause the counts of other mutation types to change. The number of genes passing the current filter threshold may also change. This is because the reduced number of mutation types decreases the qualified mutations counted for each gene. For example, if the current threshold is 3 and there is a gene X that are mutated in three samples with the mutation type being Missense, Silent and Missense in each sample. Suppose the Silent mutation

type is unchecked, then this gene no longer meets the threshold of being mutated in at least 3 samples and the number of genes passing this threshold will decrease by one. On the other hand, as this gene is dropped from the top mutated gene list, the count of total missense mutations for genes in this list will decrease by two.

The Visualization View

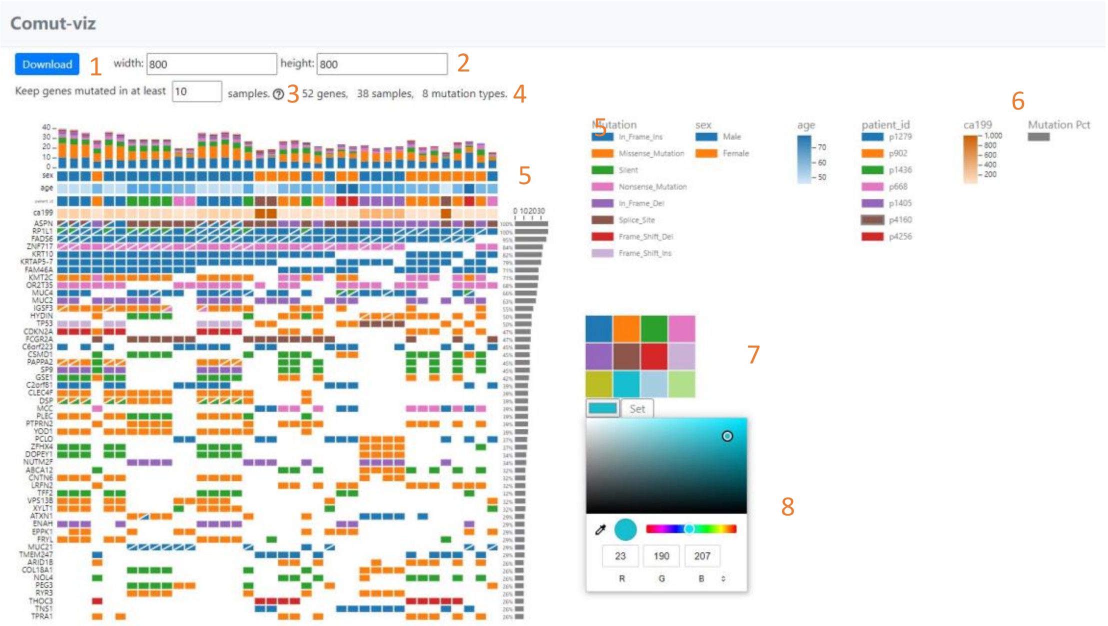

Figure S3. The visualization view with the “example 5k” data loaded.

In creating a comutation plot, three features are constantly adjusted: the width and height, the number of genes to keep and the colors. Comut-viz (Figure 3) provides two input boxes (2) to adjust the width and height, a filter to tune the number of genes (3) and a color picker (7) to customize the colors. The statistic information (4) of the plot is updated with each filtering. The app implements two kinds of legends (6): categorical legends for string data and gradient legends for numerical data. Clicking on a legend will open up the color picker

(7). It consists of a color palette of 12 distinct colors and a color box that displays the current color. Users can either choose a color in the palette or create a customized color by clicking on the color box. The color selection panel (8) may look different in different browsers. The figure shows the color selection panel in the chrome browser. After a color is selected, click on the set button to apply it to the plot and the legend. They will be updated instantly. Mouse over a gradient legend will show the range of its values. Clicking on the "Download" button (1) downloads the plot and the legend as two separate SVG figures that can be edited in vector graphics editors such as Adobe Illustrator or Inksc

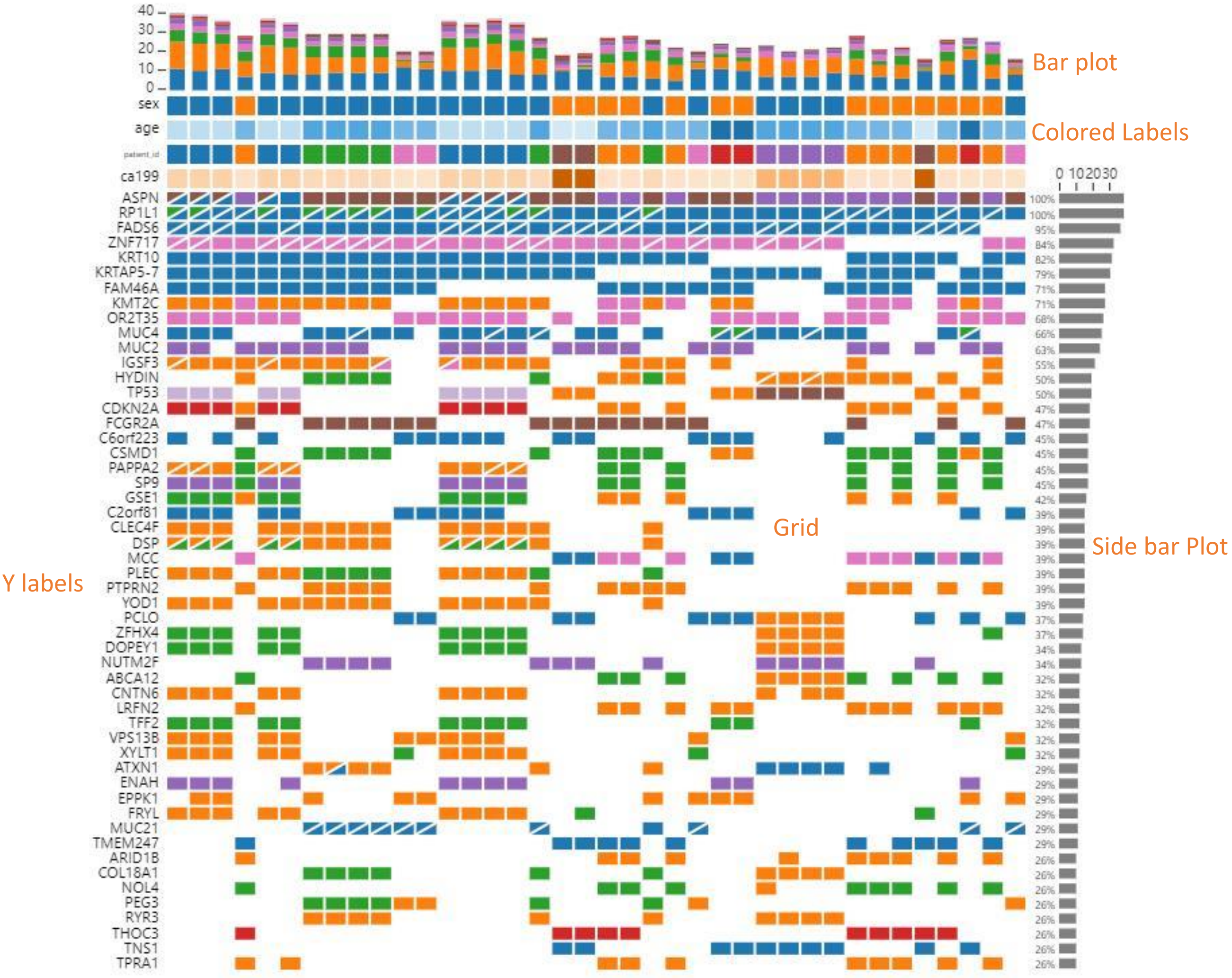

Figure S4. The components of a comutation plot.

The plot (Figure 4) is made up of a grid, a top bar, a side bar, colored labels and Y labels. In the grid, mutations are drawn as rectangles and colored by mutation type. If a gene has more than one mutation in a sample, two triangles are drawn to indicate multiple occurrences. The types of mutations will be represented first and the number of mutations second. That is, if there are two types of mutations occur in the same gene with each type occurring multiple times, two triangles of different colors will be drawn. If there are more than two types of mutations occurred in a gene, a special colored rectangle with the label “multiple” will be drawn. Mouse over any shape on the plot reveals detailed information. The colored labels will be drawn only if a sample metadata is uploaded.

Implantation Flowchart

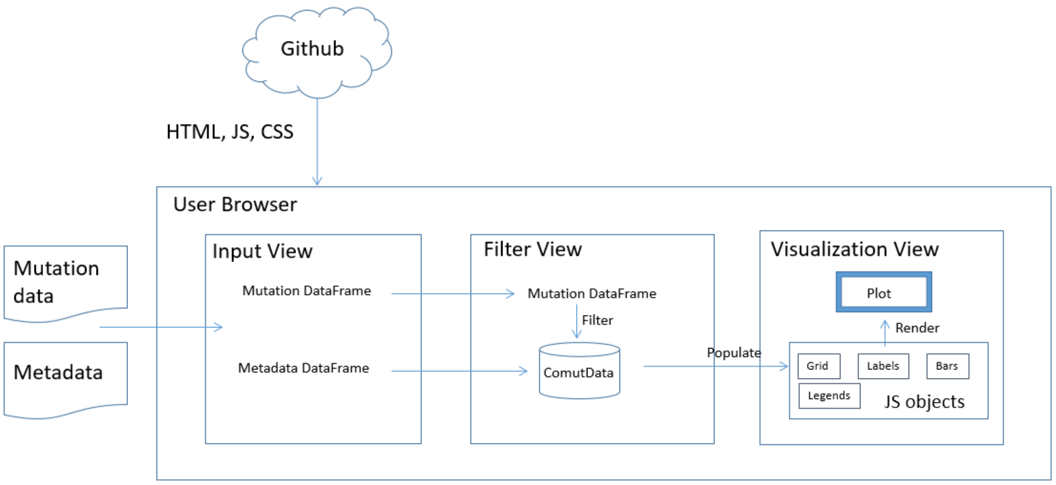

Figure S5. A schematic flowchart illustrating how Comut-viz works under the hood.
